# Oncostatin M Receptor as a Therapeutic Target for Radioimmune Therapy in Synovial Sarcoma

**DOI:** 10.3390/ph15060650

**Published:** 2022-05-24

**Authors:** Sarah McCollum, Austen Kalivas, Matthew Kirkham, Kaden Kunz, Jeffrey Okojie, Adriene Pavek, Jared Barrott

**Affiliations:** Department of Biomedical and Pharmaceutical Sciences, College of Pharmacy, Idaho State University, Pocatello, ID 83209, USA; luelsara@isu.edu (S.M.); amkalivas@gmail.com (A.K.); kirkmatt@isu.edu (M.K.); kadenkunz@isu.edu (K.K.); jeffreyokojie@isu.edu (J.O.); adrienepavek@isu.edu (A.P.)

**Keywords:** synovial sarcoma, sarcoma, oncology, radioimmune therapy, RIT, OSMR

## Abstract

Synovial sarcoma (SS) is a pediatric muscle cancer that primarily affects adolescents and young adults and has few treatment options. Complicating the treatment of synovial sarcoma is the low mutational burden of SS. Inflammatory pathways have been identified as being upregulated in some SS, leading to the discovery of upregulated oncostatin M receptor (OSMR). It was found that OSMR is upregulated in SS by RNAseq analysis and quantitative PCR, highlighting its potential in the treatment of SS. Also, OSMR is upregulated in mouse models for synovial sarcoma as demonstrated by western blot and immunohistochemistry, and the protein is present in both primary and metastatic sites of disease. Using a radioimmune therapy drug model, targeted therapy was synthesized for use in OSMR expressing SS and it was demonstrated that this drug is stable, while capable of efficient OSMR binding and isotope capture. Finally, this antibody conjugate exhibited ideal pharmacokinetics and targeted sites of disease in our mouse model and was taken up in both primary and metastatic diseased tissue. This suggests OSMR as an ideal target for therapy and this radioimmune therapy provides a novel treatment option for a disease with few therapy choices.

## 1. Introduction

Synovial sarcoma (SS), sometimes named synovial cell sarcoma, is a soft tissue malignancy affecting adolescents. While soft tissue sarcomas represent less than 1% of all cancers [1], and synovial sarcoma represents 5–10% of all soft tissue sarcomas [2], those affected by this disease have few options for treatment. In 1993, Ladenstein et al. described the benefits of adjuvant chemotherapy with doxorubicin and cyclophosphamide-based treatment after surgical resection as opposed to surgery only, and this has been the standard of therapy since then [3]. Currently, ifosphamide is the preferred cyclophosphamide-based therapy and this is often given in combination with doxorubicin [4]. Despite 30 years passing and the advancements made in the field of oncology, few alternatives to replace this harsh therapy regime have been made, highlighting the need for advancement in this field. 

SS has a 5-year survival rate of 60% which is relatively high [5], however, the survival rate is greatly dependent on the metastatic status of the patient. One study found that patients with local disease have a higher 5-year survival rate of 69% and a 10-year survival rate of 51%, while patients with metastatic disease had a 5-year survival rate of 52% and a 10-year survival rate of 9% [6]. Unfortunately, studies have shown that approximately half of the patients diagnosed with SS will develop metastasis within 5 years [7].

Caused by a single translocation mutation between chromosomes 18 and X [8], SS has a relatively low mutational burden [9,10] making targeted therapy difficult to develop based on genetic mutations. Despite the scarcity of mutations in SS, we have identified oncostatin M receptor (OSMR) as a cell surface receptor that can be overexpressed in both metastatic and non-metastatic SS. OSMR is a member of the gp130 cytokine receptor family and receives the ligand oncostatin M (OSM). This receptor is composed of the subunit gp130, which characterizes the family, and OSMRβ [11]. OSMR is known to have roles in hematopoiesis across species [12]; however, it has been shown that OSMR knock-out mice, while having abnormal blood count levels, are viable and without significant abnormalities [13]. This, along with the unique overexpression of OSMR in SS, suggests the receptor as a viable target for therapy. 

While small molecule inhibitors have been the backbone of targeted therapy in the field of oncology, occasionally they can lead to resistance and pleiotropic off-target effects. OSM is known to interact directly with the gp130 subunit of the OSMR receptor with a low affinity. After this association occurs, the OSMRβ subunit enters and associates more strongly with the OSM-gp130 complex [14]. Due to this mechanism, it is possible for OSM, or any OSM-like small molecule inhibitors, to bind with low affinity to any of the gp130 cytokine receptors and induce off-target effects resulting in toxicities. In order to develop an effective treatment targeting this receptor, it would need to be specific to the OSMRβ subunit. One way to increase the specificity of targeted therapies is to utilize antibodies. These biologics can be targeted to specific epitopes of proteins, eliminating the possibility of any toxicity inducing off-target effects. 

An emerging field in biological pharmaceuticals has been radioimmune therapy (RIT), which uses antibodies to direct radiation directly to the site of disease [15,16]. After systemic injection of an RIT, the drug then circulates throughout the body finding all sites where the antibody can bind. This means that the drug will not only have high specificity for its target but will also be able to find all sites of metastasis [17]. Once bound to the malignant cells, the radiation can kill the bound cell as well as the surrounding cells, regardless of their epitope expression [18]. Theoretically, this will result in lower rates of drug resistance and refractory disease. Furthermore, the effects of radiation on peripheral cells which survive the radiation but experience enough exposure to induce mutational effects could lead to increased antigenicity of the cells, causing an immune response against them [19]. 

In this study, we develop an RIT therapy that targets OSMR and shows that the OSMR protein is present in substantial quantities in a subset of synovial sarcoma and that OSMR expression is independent of the metastatic state. We also show that the RIT is stable and is targeted to sites of disease sufficiently through OSMR targeting, and that, while tumors with higher OSMR expression internalize the drug more efficiently, even tumors with extremely low amounts of OSMR expression are still targeted effectively.

## 2. Results

### 2.1. OSMR Is Present in Sufficient Quantities for Therapeutic Targeting

**Figure 1:**
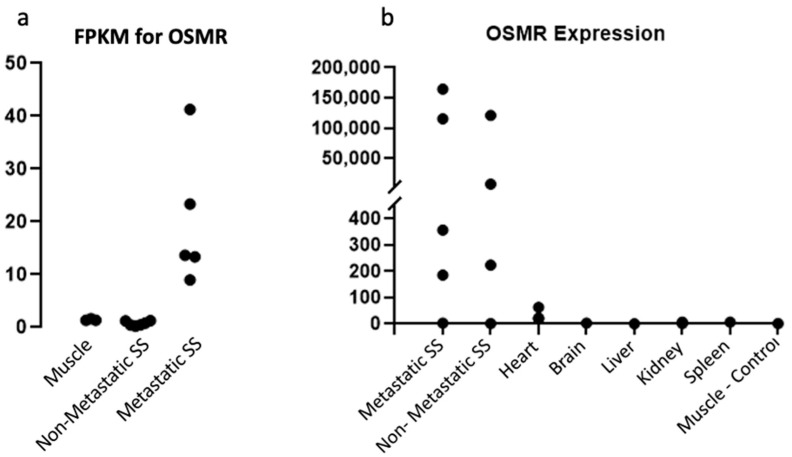
OSMR Expression is Independent of Metastatic Status. (**a**) RNAseq data of metastatic and non-metastatic tumors as well as normal muscle tissue shows a correlation between metastatic status and OSMR expression. (**b**) RTqPCR shows no such correlation and that both states can have high or low OSMR expression. Low expression of OSMR was also noted in the heart.

In our investigation into viable targets for SS treatment, RNAseq data were obtained and analyzed looking for upregulated targets in the inflammatory pathways based on evidence that SS showed signs of increased inflammation [20]. OSMR was identified as an ideal target due to it being a membrane-bound, extracellular protein as well as its specificity to malignant tissue. It was observed that OSMR was expressed at high levels in metastatic SS tissue whereas there was no expression in the normal muscle tissue (Figure 1a). Furthermore, the metastatic SS mouse model expressed OSMR at a 22.4-fold increase over the non-metastatic model, suggesting that an OSMR targeted treatment might be more effective against aggressive tumor phenotypes.

In order to confirm the aberrant expression of OSMR in tumor tissue, we first performed RTqPCR. Using both a metastatic and a non-metastatic mouse model, three mice from each group were selected at random and the RNA expression levels were measured. A sample of non-malignant muscle tissue was used as a control. Interestingly, it was found that the expression of OSMR varied greatly between samples seemingly independent of metastatic status (Figure 1b). Further repetition of this experiment with other mice showed the same pattern of great variation in OSMR expression independent of metastatic status. This suggests that the RNAseq data randomly selected mice from the metastatic cohort which were high in OSMR expression, while the non-metastatic samples happened to have low expression levels, resulting in the apparent correlation. 

**Figure 2:**
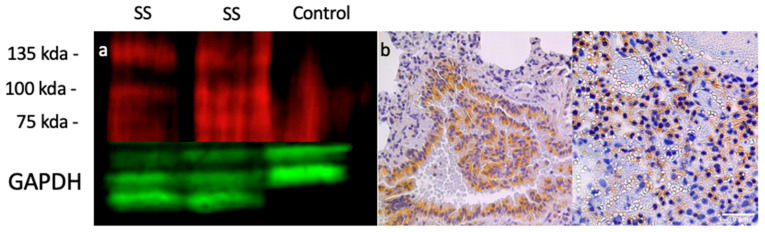
Protein expression of OSMR. (**a**) Western blot using immunofluorescent tags. OSMR is in red and control GAPDH is in green. Three bands were detected corresponding to the OSMR receptor (135 kDa), IL-31 receptor (100 kDa), and the OSMR subunit alone (75 kDa). Samples 1 and 2 are metastatic tumor samples, sample 3 is normal muscle tissue. (**b**) Immunohistochemistry staining of lung metastasis (left) and primary tumor (right). OSMR expression appears brown in tissue samples.

To evaluate the potential for off-target toxicities of an anti-OSMR treatment, we also measured the OSMR expression of various organs. While the brain, liver, and kidneys had minimal OSMR expression, it was noted that there are low levels of OSMR expression in the heart (Figure 1b). However, this expression is much lower than that of the OSMR-high tumors and while the risks of cardio-toxicity induced by an anti-OSMR drug should be investigated, we believe that the risk is minimal to patients with a high OSMR expression tumor profile. To further explore these expression patterns, western blotting was performed to measure the presence of OSMR protein in these same samples (Figure 2a). Three bands of the OSMR subunit were detected, which correlate to the OSMR receptor at 135 kDa, which is composed of the OSMRβ subunit and a gp130 subunit; the IL-31 receptor at 100 kDa, which is composed of the OSMRβ subunit and the IL-31 RA subunit; and the OSMRβ subunit alone at 75 kDa. As with the RTqPCR data, the OSMR expression appeared to be independent of metastatic status as both sample groups had great variation in OSMR expression. Furthermore, the protein expression levels coincided with the RNA expression levels, further confirming the hypothesis that the RNAseq data randomly selected mice, which showed a correlation between OSMR expression and metastatic status. However, while the tumor samples had varying degrees of OSMR expression, the normal muscle tissue showed no expression indicating that OSMR is a viable target for therapy regardless of metastatic status. These data also highlight the difficulty in predicting which patients will respond well to an anti-OSMR therapy, as aggressive tumor type is not an indication of OSMR expression.

Immunohistochemistry staining for OSMR protein was also performed in both primary tumor samples and lung metastatic samples (Figure 2b). We found that OSMR is expressed in both primary and metastatic sites of disease and is found distributed throughout the malignancy. This indicates that an anti-OSMR therapy would be effective at controlling tumor growth at both primary and metastatic sites.

### 2.2. Anti-OSMR Drug Design

**Figure 3:**
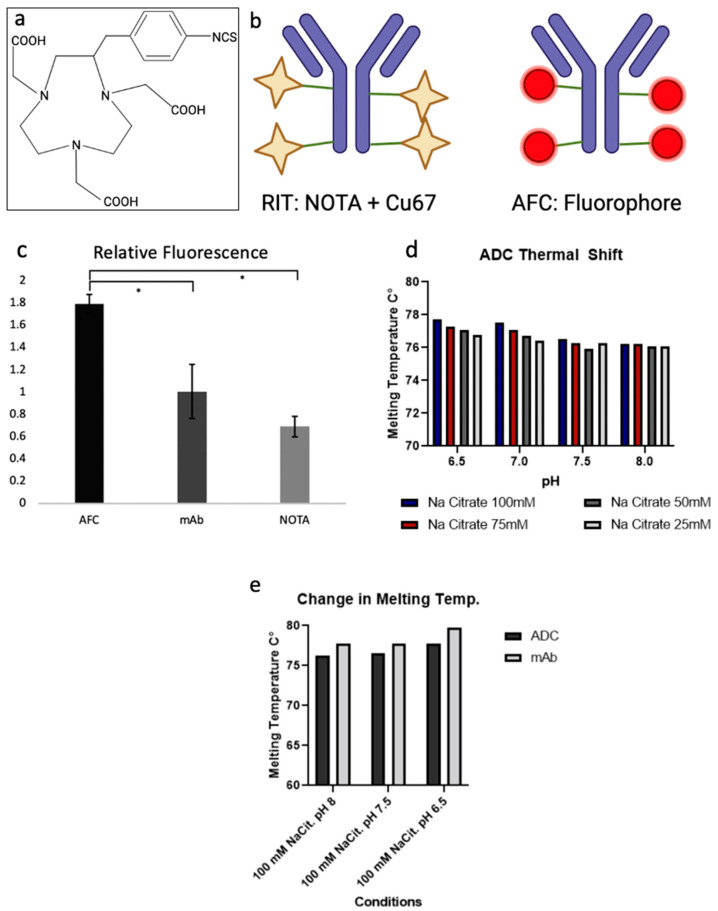
Anti-OSMR ADC Design and Characterization. (**a**) Chemical structure of p-SCN-Bn-NOTA. (**b**) Illustration of the Radioimmune Therapy (RIT) and Antibody-Fluorescent Conjugate (AFC). (**c**) Relative fluorescence of the AFC, monoclonal antibody, and chelator measured by sandwich ELISA. * *p*-Value < 0.05. (**d**) Melting temperature of the RIT in various conditions, the conjugate was found to be very stable. (**e**) Change in melting temperature of the RIT as compared to the unconjugated monoclonal antibody.

In order to achieve high specificity for the treatment target and eliminate side effects caused by off-target effects, an anti-OSMR monoclonal antibody was selected as the main structure of this therapy. Next, the literature regarding multiple chelators was analyzed to identify which would have the best stability, binding ability, and copper capture rate. Based on research comparing bifunctional chelators performed by Cooper et al. [18], we considered multiple chelators including p-SCN-Bn-DOTA, p-SCN-Bn-NOTA, and sar-CO2H. However, we ultimately decided to move forward with the p-SCN-Bn-NOTA chelator, referred to hereafter as NOTA, due to its high serum stability, efficient radio-labeling, and ideal metabolism (Figure 3a). Due to the added challenge of performing pre-clinical studies with a radioactive agent, we also developed an Antibody-Fluorescent Conjugate (AFC), in which the chelator was replaced with the fluorescent molecule, Alexa Fluor 647, which could be detected and used as a measurement of the presence of the conjugate in a safer manner (Figure 3a). This fluorophore binds to the same residues as NOTA and should mimic the properties of the conjugate without being radioactive, and is measurable through fluorescent excitation. 

A sandwich ELISA binding assay was performed to assess the ability of the conjugate to bind to its target OSMR receptor post conjugation and to ensure that the addition of a moiety to the lysine residues of the antibody did not interfere with binding. To do this, the AFC was applied to a sandwich ELISA containing OSMR protein, and binding was measured through fluorescent excitation. We found that the AFC was capable of binding to the protein showing that the attachment of groups to the lysine residues of this antibody does not interfere with the binding capabilities of the drug (Figure 3c).

Next, the stability of the ADC was tested through a thermal-shift assay. This assay was performed in the absence of radioactive materials due to the ^67^Cu limited effects on the stability of the antibody. Samples of the ADC were placed in sodium citrate buffer at varying concentrations and pH to determine which conditions provide the most stable environment. The rate of denaturation of the proteins was measured through the detection of Sypro Orange fluorescent dye. It was found that the ADC was most stable in sodium citrate at a concentration of 100 mM and a pH of 6.5; (Figure 3d), however, it was also found to be stable under a variety of different conditions. We determined that the conjugation of the NOTA chelator did not greatly affect the stability of the antibody, with only a minimal decrease in the stability compared to that of the native antibody (Figure 3e).

**Figure 4:**
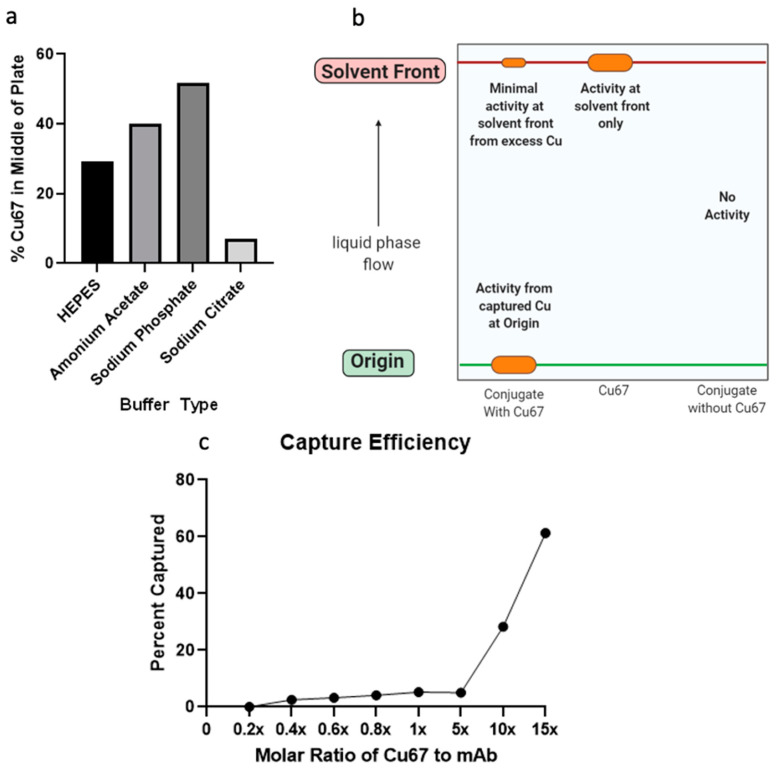
Copper Capture and Stability of the anti-OSMR RIT. (**a**) Results of testing multiple buffer types for best definition achieved on TLC plate. Best definition was measured as the conditions with the least amount of radioactivity in the middle of the TLC plate. (**b**) Schematic for how TLC tests were performed. (**c**) Results of copper capture rate experiments, capture efficiency was measured at varying molar ratios of ^67^Cu to OSMR mAb.

To confirm the ability of the ADC to efficiently capture ^67^Cu ions, thin layer chromatography was utilized. Because the literature is variable in which buffers would be best for such an experiment, multiple buffers were tested, and the definition achieved on the plate under different conditions was measured. We found that a Sodium Citrate buffer provided the best results (Figure 4a). After allowing the ADC to associate and capture copper isotopes, samples were placed on iTLC plates, a glass microfiber paper with silica gel. After elution of the samples, the plates were cut in half and the activity of the free copper at the top of the plate was compared with the activity of the captured copper at the bottom of the plate (Figure 4b). We found that the ADC was able to capture ^67^Cu somewhat efficiently and that the capture rate was concentration-dependent requiring an excess of ^67^Cu (Figure 4c).

### 2.3. Pharmacokinetic Studies of Antibody-Fluorescent Conjugate

**Figure 5:**
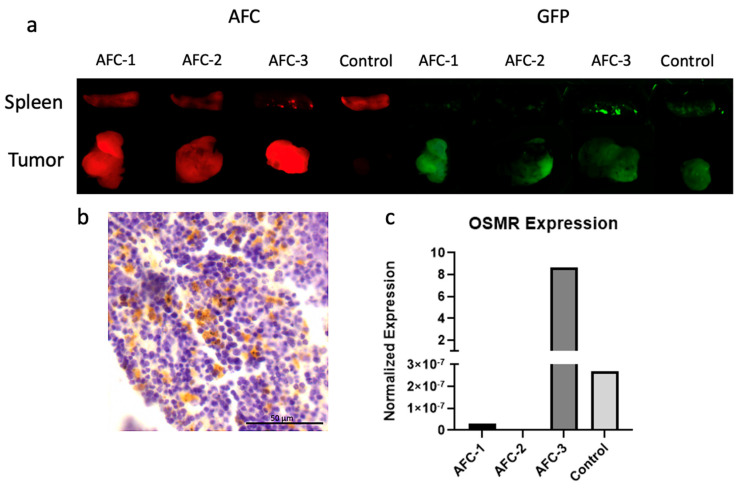
Pharmacokinetics of the AFC and the Correlation to OSMR Expression. (**a**) Fluorescent imaging of mouse spleens and tumors after treatment with AFC. Samples 1–3 received AFC treatment and sample 4 received saline control. AFC appears red (Left) and SS tissue appears green due to GFP (Right). (**b**) Immunohistochemistry of spleen metastasis for GFP from AFC-3 sample. GFP presence indicates sites of disease and appears brown. (**c**) RTqPCR data showing expression levels of OSMR. AFC and Control samples correlate to Figure 5a.

The pharmacokinetics of this newly developed drug were assessed using the metastatic mouse model aforementioned. Four mice were selected which had received a previous TATCre injection and had developed tumors of sufficient size. The AFC was injected intravenously through the tail vein and mice were harvested 48 h later. The mice were dissected and the tumors along with kidneys, liver, spleen, lungs, and heart were harvested and imaged through nIR fluorescence. Because the mouse model has a genetic modification that induces the expression of Green Fluorescence Protein (GFP) in SS cells, the malignancies could be easily observed, and some areas of metastasis were noted in the spleen of one mouse (Figure 5a). Next, imaging of the AFC was performed, and it was noted that there was exceptional targeting of the drug to sites of disease, with no drug seen accumulating in significant quantities in any other areas (Figure 5a). It was also noted that the drug not only localized to the primary tumor site but was also present in the sites of metastasis. Outside of the sites of disease, no accumulation of the AFC was seen in any other organs (Supplementary Materials Figure S1) These results indicate that an anti-OSMR antibody is an effective targeting strategy for therapy in SS.

IHC was performed on these samples to confirm the presence of OSMR protein, and OSMR expression was seen in the spleen in which metastasis was noted through fluorescence detection (Figure 5b).

Finally, we performed RTqPCR on these samples to measure the expression of OSMR relative to each other as well as normal muscle tissue. We found that OSMR expression was correlated to the level of ADC fluorescence visible in the tumors (Figure 5c). It was also noted that the drug had high specificity for sites of disease despite the OSMR expression being relatively low in some samples.

## 3. Discussion

Synovial sarcoma remains a disease in which there are few treatment options available to patients. The benefit of targeted therapy has been made evident in many other malignancies by its reduced side effects and improved tolerability [21,22,23]. In recent years, a handful of targeted therapies have been FDA approved for the treatment of soft tissue sarcomas [24], a classification under which SS falls. These advancements are greatly needed in the treatment of SS, however, as with many targeted therapies, there still exist the challenges of resistant and refractory disease [25]. Furthermore, these therapies have yet to become a standard first-line therapy in the treatment of synovial sarcoma, in part due to the rarity of the disease and the difficulty of creating a robust study size [26]. Despite this challenge, a few studies have been able to obtain robust study sizes. The EpSSG NRSTS 2005 study analyzed 150 cases of SS and evaluated their response to both chemotherapy and radiotherapy. The COG ARST0332 study was also able to obtain a large sample size of 138 patients and evaluate their response to chemotherapy and radiotherapy. Another factor that contributes to the difficulty of finding a targeted therapy to replace the current first-line treatment of doxorubicin is the low mutational burden of synovial sarcoma [10]. For this reason, increasing the number of treatment options for synovial sarcoma would benefit SS patients. Here, we detail the overexpression of OSMR in synovial sarcoma and highlight its potential as a target for therapy. 

Measurement of RNA levels and expression as well as protein expression in multiple tumor types has revealed that OSMR is overexpressed in some synovial sarcoma tumors and that the expression level is abundant enough for targeted therapy (Figure 1). Expression was also noted to be present in both the primary and metastatic sites of disease (Figure 2). OSMR, being a transmembrane receptor with an extracellular portion, is an ideal target for immunoconjugate therapy [27]. However, it was noted that OSMR is expressed at low levels in the heart, indicating the potential for cardiotoxicities (Figure 1b). We feel that the level of OSMR expression in the heart is minimal and should still allow for effective anti-OSMR treatment without significant toxicities, but plan to further research this potential side effect. Also warranting further research is the copper capture rate of our RIT (Figure 4c). While we have shown that this drug is capable of isotope capture, our current efficiency rates are below that which is seen in the literature and should be improved upon [28,29]. 

The OSMR expression profile has been found to be somewhat unpredictable and unrelated to metastatic status or tumor phenotype, making it difficult to predict which patients would be good candidates for an anti-OSMR RIT. Fortunately, the RIT drug design provides a way to measure the expected response to therapy with minor adjustments. Rather than using the beta-emitting ^67^Cu ions in the RIT, they can be replaced quite easily with gamma-emitting ^64^Cu ions, which can then be detected by PET scan after administration to the patient [29]. In doing this, the locality of the drug and its specificity for the sites of disease can be determined prior to administration of the tissue-damaging RIT. Furthermore, this provides a novel theranostic agent which can be used to locate sites of metastasis and track patient progress and response to therapy [30]. This technique will have little impact on the patient and will prevent the administration of this drug in patients with low OSMR expression who may experience poor drug targeting. This is especially important as our data show that it is difficult to predict OSMR expression based on phenotype (Figure 1). 

RIT has the potential to overcome the development of refractory disease due to inducing cell death through a non-specific mechanism of proximity to radiation rather than inhibiting a specific metabolic pathway [31]. It is well known that most tumors have a heterogenous microenvironment, and therefore, even a tumor with high OSMR expression is likely to have a few malignant cells which do not have OSMR expression [32]. These cells are still susceptible to death through this therapy, as they only need to be near the RIT, rather than directly bound to it. In this way, the risk of refractory disease development as a result of treatment is expected to be decreased when compared to conventional therapies. 

Another benefit to this model of therapy is that it is not specific to synovial sarcoma, but rather it is indicated for any malignancy which exhibits an overexpression of OSMR. Because the antibody is specific for OSMR, which has been found to be overexpressed in multiple cancer types [33], this therapy is capable of treating multiple types of cancer regardless of their classification. Due to the conserved basic structure of all antibodies, it is also a potential strategy to alter the target of the monoclonal antibody with which the ADC is composed of. This would require little alteration to the methods and should not cause any large changes to the results of treatment. 

While RIT offers a new approach in which many of the challenges of cytotoxic therapy are circumvented, it too has its downfalls. One difficulty of RIT, as with all targeted therapies, is ensuring that the target is specific enough for effective reduction in malignancy without off-target effects. Because protein expression varies from tumor to tumor and patient to patient, there is always the chance that a targeted therapy performs poorly in a subset of the population [34]. The use of ^64^Cu in this RIT provides a safeguard against the treatment of this subset and should prevent excessive toxicities [35]. Another challenge to RIT is the short half-life as a result of the radioactive isotopes along with the protein structure. The time available between production and administration is limited making the logistics of production challenging. However, a few FDA radioactive drug compounds, such as Xofigo (^223^Ra dichloride), have made their way into clinics, showing that this challenge can be overcome [36]. 

We believe that the therapeutic strategy of using OSMR targeting RIT to treat both metastatic and non-metastatic OSMR-expressing synovial sarcoma can provide patients who have few treatment options a better-tolerated therapy, as well as a novel diagnostic tool. We show that OSMR is expressed in substantial quantities in a subset of synovial sarcoma, and while this expression can be difficult to predict from a phenotypic standpoint, we also provide a solution to evaluating a patient’s predicted response to therapy through the use of theranostic ^64^Cu imaging. We have shown that an anti-OSMR RIT can be developed and is able to bind to the OSMR protein and believe that further research is indicated. 

## 4. Materials and Methods

### 4.1. Mouse Models

Using the metastatic synovial sarcoma mouse model; Rosa26-LSL-SS18-SSX2/SS18-SSX2;Pten^lox5/lox5^ [20], mice were selected at 4 weeks of age and injected subcutaneously with TATCre protein (Excellgen, Rockville, MD, USA. Cat.# EG-1001) in their left hind limb. Both male and female mice were used. Mice were monitored for signs of tumor development. All protocols were approved by the Idaho State University’s institutional animal care and use committee protocol 775.

### 4.2. RNAseq

Data were acquired using accession number GSE81476. [20].

### 4.3. Slide Preparation

Tissues were fixed in 4% Paraformaldehyde in PBS for 24 h, then washed with PBS to remove any remaining Paraformaldehyde. Tissues were embedded in Paraplast X-TRA paraffin wax (Sigma-Aldrich, St. Louis, MO, USA. Cat.# P3808-1KG) and chilled prior to sectioning. Tissues were sectioned using a Leica Histocore Autocut microtome.

### 4.4. Immunohistochemistry

Immunohistochemistry (IHC) was performed using the Ptenlox5/lox5;SSM2/SSM2 metastatic mouse model. Tissue was stained using a rabbit anti-human/mouse polyclonal antibody targeting either Oncostatin M Receptor (OSMR) (ABclonal, Woburn, MA, USA. Cat.# A6681) or eGFP (Thermo Fisher, Carlsbad, CA, USA. Cat.# CAB4211). All other reagents were obtained from the Pierce Peroxidase Detection Kit (Thermofisher, Carlsbad, CA, USA. Cat.# 36000) and protocol was followed according to the manufacturer’s protocol. Briefly, a goat anti-rabbit secondary antibody was applied to the tissues, followed by an anti-goat strep-HRP tertiary antibody. Samples were counterstained with hematoxylin, dehydrated, and mounted. Slides were analyzed through a Leica DM6B widefield microscope and imaged with the attached Leica DFC450-C digital camera. 

### 4.5. Western Blot

Western blotting was performed on tumor samples from the metastatic mouse model described above, as well as the non-metastatic mouse model Rosa26-LSL-SSM2/SSM2 [37]. Roughly 50 mg of tissue was obtained from each mouse. Samples were chemically digested with RIPA buffer (0.15 M NaCl, 0.05 M tris-cl, 1 M NP-40, 0.5 M Sodium Deoxycholate, 0.1 M SDS) and homogenized mechanically. The protein concentration of each sample was measured using the Qubit 3 Fluorometer (Thermofisher Invitrogen, Grand Island, NY, USA. Cat.# Q3321) and samples were prepared by loading 430 μg of protein per sample. This high protein concentration was used due to the low concentration of OSMR per sample and 430 μg was selected based on the sample with the lowest concentration. Samples were run on a Novex 4–12% Tris-glycine gel (Thermofisher, Carlsbad, CA, USA. Cat.# XP04120BOX) at 160 volts until proper separation of bands had been achieved. Next, the samples were transferred from the gel to an Immun-Blot^®^ LF PVDF membrane (Bio-Rad, Hercules, CA, USA. Cat.# 162-0263) and processed for imaging using an anti-OSMR rabbit polyclonal antibody (ABclonal, Woburn, MA, USA. Cat.# A6681) as a primary antibody and fluorescently labeled using an anti-rabbit IgG-CFL 680 (Santa Cruz Biotechnology, Eugene, OR, USA. Cat.# sc-516252) as a secondary antibody. Control GAPDH was conducted using an anti-GAPDH goat polyclonal antibody (Sigma-Aldrich, St. Louis, MO, USA. Cat.# PLA0302) as the primary antibody and fluorescently labeled using an anti-goat CFL-790 labeled secondary antibody (Santa Cruz Biotechnology, Eugene, OR, USA. Cat.# sc-516246) Imaging was performed on an Azure c600 system and the fluorescent label was excited at 790 nM and visible at 800 nM appearing red in the image. The GAPDH fluorescent label was excited at 680 nM and was visible at 700 nM appearing green in the image.

### 4.6. RTqPCR

Reverse transcriptase quantitative PCR (RTqPCR) was performed on samples obtained from both metastatic and non-metastatic models, (see above for mouse model description). RNA was extracted from samples using a Quick-RNATM MiniPrep Plus kit (Zymo Research, Irvine, CA, USA. Cat.# R1058) and cDNA was made using Qscript cDNA Supermix (Quantabio, Gaithersburg, MD, USA. Cat.# 95048). cDNA was amplified and measured using the Eppendorf Mastercycler Realplex2. Data were analyzed using a delta CT calculation.

### 4.7. ADC Preparation

#### 4.7.1. Antibody Purification

The ADC was made using a monoclonal anti-OSMR antibody (Sino Biological, Beijing, China. Cat.# 11226-R002). This anti-OSMR mAb was selected based on its specificity for the extracellular portion of the OSMR protein, and being a product of rabbits allows us to test this antibody in both mouse and human samples. It was purified by human OSMR affinity chromatography with a protein A and antigen affinity column to be of high quality. Our own studies show that this product is stable until high temperatures (Figure 3e) and binds to OSMR (Figure 3c). This specific product has been used successfully in on-cell western assays [38] and in western blot analysis [39]. The antibodies were purified to eliminate the presence of any ions or contaminants. This was done using a Slide-A-LyzerTM dialysis cassette (ThermoFisher, Irvine, CA, USA. Cat.# 66373). Briefly, EDTA was added to the antibody sample and placed in the cassette. The cassette was then incubated in Sodium Citrate buffer 0.1 M with a pH of 6.5. The sample was removed and concentrated using a Vivaspin 500 protein concentrator (Sartorius, Goettingen, Germany. Cat.# VS0191) and centrifuged at 10,000 g and 4 °C for 1 h. Once protein was concentrated the concentration was measured using the Qubit 3 Fluorometer (Thermofisher, Wilmington, DE, USA. Cat.# Q33216). 

#### 4.7.2. Chelator Conjugation

After the anti-OSMR monoclonal antibody had been purified, it was incubated at room temperature in the presence of a 40 molar excess of NOTA chelator (Macrocyclics, Plano, TX, USA. Cat.# B-605) and the reaction was allowed to proceed for 2 h. Then the reaction was moved to 4° C and allowed to incubate overnight. Once the conjugation was complete, the excess chelator was removed using a Vivaspin 500 protein concentrator (Sartorius, Goettingen, Germany. Cat.# VS0191).

#### 4.7.3. Radiolabeling of ADC

The ADC was labeled using ^67^Cu provided to us by the Idaho Accelerator Center. Capture was performed by mixing copper with the conjugate and incubating for 20 min at 27 °C in 0.1 M HEPES buffer with a pH of 8.5. This experiment was designed to keep activity levels low, with a total of 37 MBq (1 mCi) of radioactive material being used for this experiment. The concentration of the antibody-NOTA conjugate was 20 pM with a molar ratio of ^67^Cu ranging from 4 pM (0.2x) to 300 pM (15x).

#### 4.7.4. AFC Preparation

The antibody was purified as described above. Antibody-Fluorescent Conjugate (AFC) was prepared using a Zip Alexa Fluor Rapid Antibody Labelling Kit (Invitrogen, Grand Island, NY, USA. Cat.# Z11235) and the manufacturer’s protocol was followed. Briefly, a 1mg/mL solution of an anti-OSMR monoclonal antibody (Sino Biological, Beijing, China. Cat.# 11226-R002) was added to a 1 M solution of Sodium Bicarbonate containing the fluorescent dye and incubated at room temperature for 15 min.

### 4.8. Murine Pharmacokinetic Studies

Mice from the metastatic model previously described were selected 10–14 weeks after receiving a TAT-Cre injection to their left hind limb. Mice were treated via a tail vein injection of AFC at 0.4 mg/kg or received a saline control. Mice were harvested 48 h later and tissues were imaged for AFC retention using the Azure C600 imager with nIR imaging properties. The AFC was excited at 650 nm and emission was detected at 665 nm. GFP was excited at 450 nm and emission was detected at 510 nm. 

### 4.9. Sandwich ELISA Binding Assay

A sandwich ELISA was performed using a Human OSMR beta ELISA kit (Novus Biologics, Centennial, CO, USA. Cat.# NBP2-68083). The plate was first treated with human OSMR beta protein by adding 100 uL of 10ng/mL. The plate was incubated at 37 °C for 90 min. The liquid was aspirated from the plate and treated with AFC 0.15 μg /uL, pure OSMR antibody 0.15 μg /uL, or PBS as a control. The plate was incubated for 1 h and liquid aspirated. Biotinylated antibody was applied to wells according to the manufacturer’s protocol and incubated at 37 °C for 1 h. The liquid was removed and plates were washed with wash buffer according to the manufacturer’s protocol. HRP solution was applied to each well and incubated for 30 min at 37 °C. Plates were washed and substrate reagent was applied and incubated for 15 min at 37 °C and protected from light. Stop solution was added to each well prior to measurement on a Varioskan LUX multimode microplate reader (ThermoFisher, Singapore, Cat.# VL0000D0). 

### 4.10. Thin Layer Chromatography

Thin layer chromatography was performed using instant thin layer chromatography plates (iTLC) to separate the compounds (Agilent, Santa Clara, CA, USA. Cat.# SGI0001) Briefly, the sample was placed at the origin and allowed to dry for roughly 5 min. Then the plate was placed in a buffer and allowed to develop. Once finished, the plate was cut into sections and the activity of each section was measured using a Ludlum 3030 sample counter. For definition experiments, the plate was cut into three pieces (top, middle, and bottom) and the activity found on the middle section was measured to determine the level of definition. For copper capture experiments, the plate was cut into two pieces and the ratio of activity on the bottom vs. the top was compared. Background radiation measurements were performed approximately every 10 min.

### 4.11. Thermal Shift Stability Assay

Unconjugated monoclonal antibody and the conjugated ADC were prepared in sodium citrate buffer at 25 mM, 50 mM, 75 mM, and 100 mM. Each concentration of sodium citrate was brought to a pH of either 6.5, 7.0, 7.5, or 8.0. Each sample condition was run in triplicate in a 96 well plate and included SYPRO Orange dye (Sigma-Aldrich, St. Louis, MO, USA. Cat.# S5692) at a concentration of 5x. The plate was analyzed using an Eppendorf Mastercycler Realplex2 where the temperature was steadily increased from 30°C to 95°C over the course of 20 min.

## Data Availability

Data is contained within the article and Supplementary Materials.

## Supplementary Materials

The following supporting information can be downloaded at: https://www.mdpi.com/article/10.3390/ph15060650/s1, Figure S1: Pharmacokinetics of AFC in Mice.
