# Supplementary material for: Oncostatin M Receptor as a Therapeutic Target for Radioimmune Therapy in Synovial Sarcoma
Source: Pharmaceuticals (Basel). 2022 May 24;15(6):650. doi: 10.3390/ph15060650 (PMC9228444; doi:10.3390/ph15060650)
Supplement: Supplementary file 1 [file pharmaceuticals-15-00650-s001.zip › pharmaceuticals-1704149-supplementary.pdf]

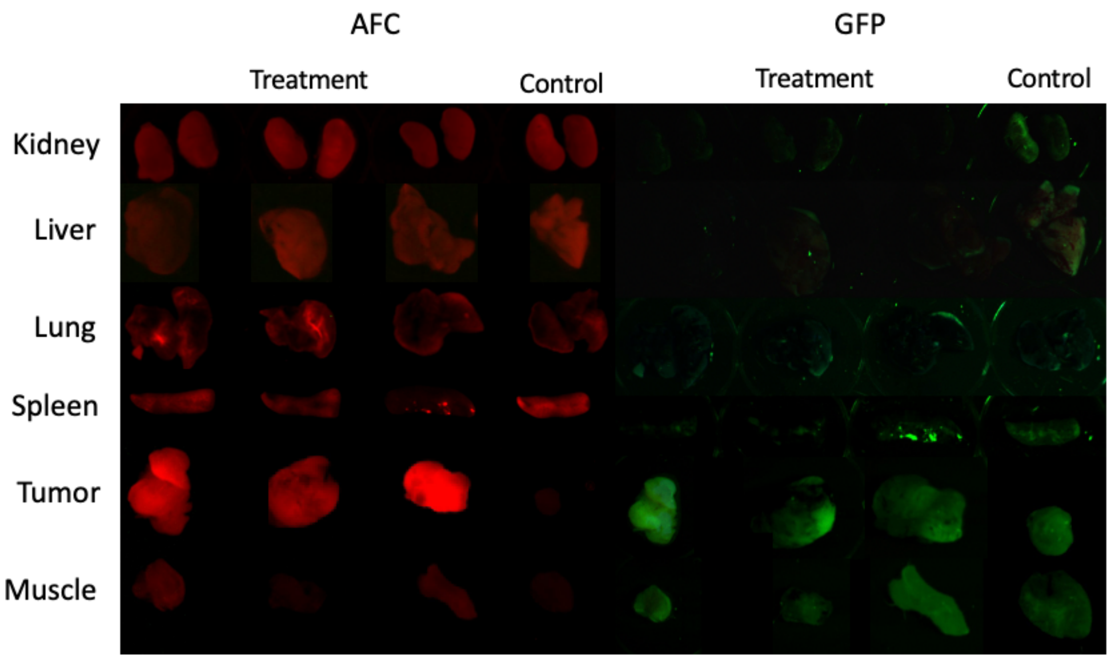

Figure S.1
Fluorescent imaging of mouse organs and tumors after treatment with AFC. Samples 1-3 received AFC treatment and sample 4 received saline control. AFC appears red (Left) and SS tissue appears green due to GFP (Right).
